# Venous Malformation in the Auricle

**DOI:** 10.3390/diagnostics12112579

**Published:** 2022-10-24

**Authors:** Junhui Jeong, Yeejeong Kim

**Affiliations:** 1 Department of Otorhinolaryngology, National Health Insurance Service Ilsan Hospital, Goyang 10444, Korea; 2 Department of Pathology, National Health Insurance Service Ilsan Hospital, Goyang 10444, Korea

**Keywords:** venous malformation, vascular malformation, auricle, external ear

## Abstract

A venous malformation is a congenital malformation of the vascular venous system. It can occur anywhere in the body, but is most common in the head, neck, and extremities. Venous malformations in the auricle are rare. A venous malformation in the auricle of a 44-year-old woman is presented.

**Figure 1:**
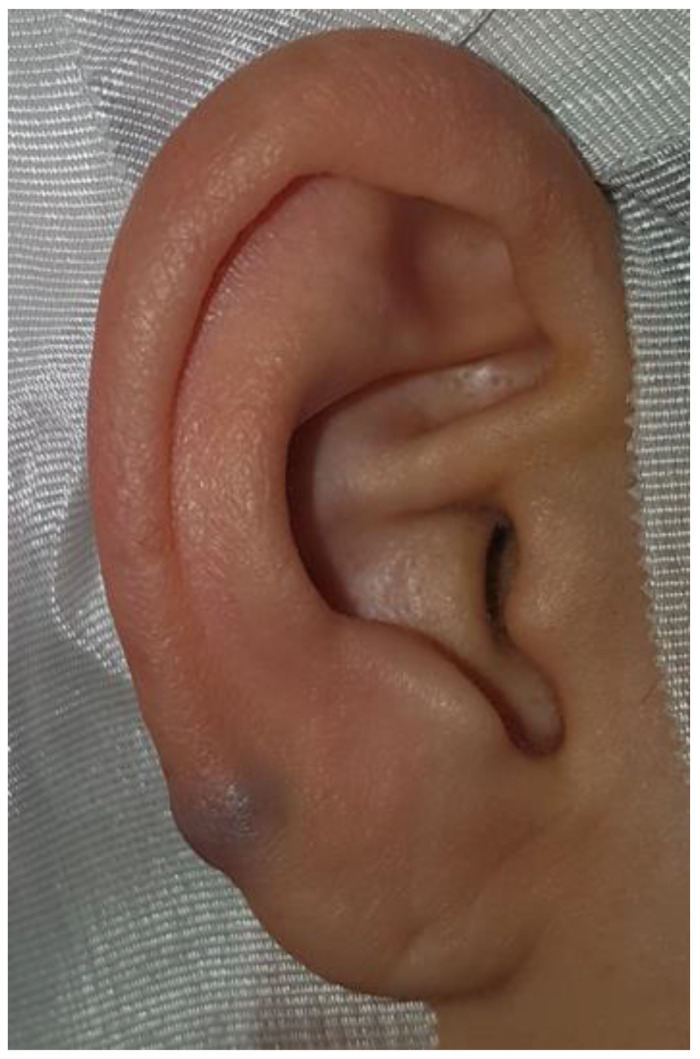
Venous malformation is a congenital malformation of the vascular venous system [1]. It can occur anywhere in the body, but is most common in the head, neck, and extremities [2]. Venous malformation in the auricle is rare and no cases have been reported. A 44-year-old woman visited the otorhinolaryngologic clinic for a mass in the right auricle that had been present for five years and had shown no recent changes in size. She had no symptoms such as pain or bleeding. On physical examination, a blue, soft cystic mass about 0.5 × 0.5 cm in size was observed at the lower helix of the right auricle.

**Figure 2:**
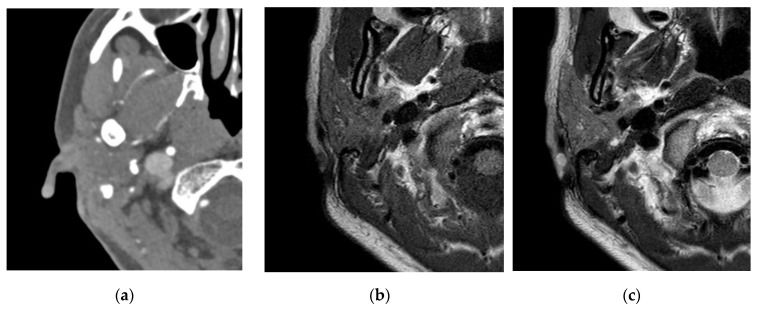
On brain computed angiography, an enhanced mass about 0.5 × 0.5 cm in size with soft tissue density was revealed at the helix of the right auricle. (**a**) Through neck magnetic resonance imaging (MRI), the mass at the helix of the right auricle was revealed with low signal intensity in T1-weighted images (**b**) and high signal intensity in T2-weighted images. (**c**) With suspicion of vascular tumor, surgical excision was planned upon patient request.

**Figure 3:**
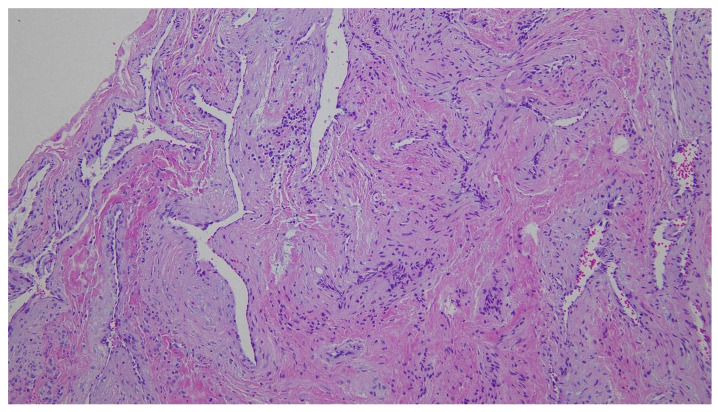
In surgery, the soft cystic mass was dissected from the overlying skin. It did not extend to the conchal cartilage and was completely removed. On histopathology, irregularly dilated venous channels with sparse smooth muscle cells without arteries were observed (hematoxylin-eosin, ×100). Thus, venous malformation was diagnosed. Six months after surgery, the wound had healed well and there was no recurrence.

Mulliken and Glowacki classified vascular lesions into two categories, hemangioma and vascular malformation, in 1982 [3,4,5]. The International Society for the Study of Vascular Anomalies classified vascular anomalies into two types, vascular tumors and vascular malformations, based on the original classification by Mulliken and Glowacki [1,2,3,6,7,8].

Vascular malformations and tumors show different growth characteristics. A vascular malformation includes venous malformation, lymphatic malformation, and arteriovenous malformation [6]. A venous malformation is a congenital lesion that occurs due to a somatic mutation and development of a dysplastic vein [2]. To the best of our knowledge, this is the first report of a venous malformation in the auricle.

The venous malformation causes a bluish to purple discoloration when it involves the skin [1]. Pain is common due to the mass effect, venous congestion, and intermittent painful thrombosis [8,9]. If local intravascular coagulation occurs, signs and symptoms of local inflammation may appear [1], and there is no palpable thrill or audible bruit [9]. In contrast, pulsation and palpable thrill are present in arteriovenous malformations due to high-flow arteriovenous shunting [7]. Arteriovenous malformations in the auricle may induce symptoms of pulsation, bleeding, and pain [4].

Venous malformations appear as isointense in T1-weighted images and hyperintense in T2-weighted images, and it is enhanced with contrast [1]. T2-weighted images from MRI can be used to differentiate vascular lesions. Hemangioma and venous malformations show a more intense enhancement than arteriovenous and lymphatic malformations. Venous and lymphatic malformations show a high signal intensity in T2-weighted images, whereas hemangioma and arteriovenous malformations show an intermediate signal intensity in T2-weighted images [10].

Treatment is not necessary in most venous malformations because they are small and asymptomatic. When it is needed, treatment is aimed for symptomatic relief and prevention of progression rather than cure in most patients [9]. The treatment of a venous malformation includes compression, anti-inflammatory agents, anticoagulants, sclerotherapy, surgery, or laser therapy [6,8]. Surgical excision is possible in well-defined lesions [6,9], but is associated with high recurrence rates [2,9].

The major difference between a vascular tumor such as hemangioma and a vascular malformation is that there is increased endothelial cell turnover on histopathology in hemangioma [2,7]. Vascular malformations rarely regress [6,9], and are associated with infection, trauma, ligation, attempted excision, and changes in serum hormone levels [5].

Though a venous malformation in the auricle is rare, it should be considered in evaluation of a cystic vascular lesion in the auricle. Surgical excision can be considered in a lesion with symptoms or that which is suspected of being other pathologies to confirm the histopathological diagnosis.

## Data Availability

The data presented in this article are available on request from the corresponding author.
